# A Clinical Case

**Published:** 1887-07

**Authors:** A. B. Eadie

**Affiliations:** Toronto, Canada


### CLINICAL CASE.

A. B. EADIE, M. D., TORONTO, CANADA.

December 13th, 1886., Mr. M. complains as follows:

I.—Pain in left side and small of back, aggravated by breathing; cannot lie on either side on account of it; must lie on the back with hips elevated by pillow.

II.—Nausea worse after meals.

III.—Dull headache across forehead.

IV.—Very thirsty.

V.—Urine clear, frequent; urgent, can't hold it.

VI.—Bowels at present open; has been much troubled with constipation.

VII.—Sleep restless; wakes by starts.

VIII.—Sweating to-day; feels hot and feverish; last night was chilly and thirsty; felt relieved after drinking.

IX.—*Feet sweat a great deal.*

X.—White, thick, painless discharge from urethra (thinks he has contracted gonorrhœa, as he has been exposed).

XI.—Feels some abdominal pain.

XII.—Had an attack of faintness this morning at half-past nine which lasted one-half hour.

XIII.—Has four ulcers on left leg between knee and ankle—one on anterior and inner aspect and three on posterior and outer, one of these being about the size of a twenty-five-cent piece and the others about that of a ten-cent piece, irregularly circular in form, very irritable, and having a thin, offensive discharge tinged with blood. They have persisted in spite of a variety of treatment since October 4th and are the special reason of this application for relief.

XIV.—He had to-day profuse epistaxis very difficult to stop.

Ferrum$^{2c}$, three powders.

December 14th.—Immediate improvement, ulcers feel better; he says they are healing.

December 17th.—Slept better last night than for months; ulcers evidently smaller and covered with laudable pus; the case progressed to complete recovery, all symptoms disappearing. Three of the ulcers healed in two weeks, the larger continuing a little longer. The remedy was repeated three times in the course of the treatment. The sweating of the feet, which I have not seen under Ferrum, was also removed.

